# Dynamic Changes in Pentraxin-3 and Neprilysin in ST Segment Elevation Myocardial Infarction

**DOI:** 10.3390/biomedicines10020275

**Published:** 2022-01-26

**Authors:** Rahel Befekadu, Magnus Grenegård, Anders Larsson, Kjeld Christensen, Sofia Ramström

**Affiliations:** 1 Department of Laboratory Medicine, Section for Clinical Immunology and Transfusion Medicine, Örebro University Hospital, 70185 Örebro, Sweden; 2 Cardiovascular Research Centre, School of Medical Sciences, Faculty of Medicine and Health, Örebro University, 70185 Örebro, Sweden; magnus.grenegard@oru.se (M.G.); sofia.ramstrom@oru.se (S.R.); 3 Department of Medical Sciences, Uppsala University, 75185 Uppsala, Sweden; anders.larsson@akademiska.se; 4 Karlstad Central Hospital, 65230 Karlstad, Sweden; Kjeld.Christensen@regionvarmland.se; 5 Department of Clinical Chemistry, Department of Biomedical and Clinical Sciences, Linköping University, 58185 Linköping, Sweden

**Keywords:** ST elevation myocardial infarction, C-reactive protein, PTX3 protein, neprilysin, thrombectomy, survival analysis

## Abstract

Pentraxin-3 (PTX3) and neprilysin have been associated with increased morbidity and mortality in chronic inflammatory disease and heart failure, but these biomarkers have been studied less in patients with ST segment elevation myocardial infarction (STEMI). We investigated the dynamic changes in these biomarkers, as well as the well-known C-reactive protein (CRP), in STEMI patients. PTX3, neprilysin and CRP were measured in samples from 165 STEMI patients, collected at the acute stage, 1–3 days after and 3 months after percutaneous coronary intervention (PCI), and from 40 healthy donors. Patient survival was followed for approximately 8 years after the PCI. As compared with samples from healthy donors, plasma levels of CRP and PTX3 were significantly increased in the acute samples and 1–3 days after PCI, but not at 3 months. CRP levels peaked at 1–3 days, while PTX3 was similarly high in both acute and 1–3 days samples. For neprilysin, no significant differences were observed at the group level. We found no significant differences when comparing patients with patent versus occluded culprit vessels or between patients having a thrombus aspiration or not. However, we found a significant reduction in survival for individuals with PTX3 above the median, both for samples collected at the acute stage and 1–3 days after PCI (*p* = 0.0001 and *p* = 0.0008, respectively). For CRP, no significant differences were observed using this approach, but patients above the reference range for healthy donors in the acute samples showed significantly lower survival (*p* = 0.0476). Conclusions: Survival analysis suggests that PTX3 might be a promising marker to predict mortality in this patient population.

## 1. Introduction

The inflammatory response has an important role during the acute phase and healing process after acute myocardial infarction (AMI) [1].

C-reactive protein (CRP) is a member of the pentraxin protein family, and increased levels of CRP were observed several years ago in plasma from patients with acute infections, when it reacted with C polysaccharide from pneumococci [2,3]. In acute inflammation, this protein is mainly synthesized by hepatocytes in response to pro-inflammatory cytokines released by macrophages and fat cells, especially interleukin-6 [3,4]. As CRP attaches to phosphocholine groups in the damaged myocardial cell membrane and activates the complement system, and also enhances phagocytosis by macrophages and activates inflammation, it has been suggested to be an important player in the pathophysiology of MI [5,6]. CRP is one of the substances found in the atherosclerotic lesion, especially in the vascular intima, where it co-localizes with monocytes, monocyte-derived macrophages and lipoproteins [2,4,7]. CRP has been extensively studied and recognized as a general inflammation marker and as an important marker for both AMI development and established coronary artery disease [8].

In the early 1990s, a new member of the pentraxin superfamily was recognized, long pentraxin-3 (PTX3) [9]. PTX3 is an acute-phase reactant that shares structural and functional homology with CRP and serum amyloid P component [10]. However, unlike CRP, which is synthesized mainly in the liver in response to interleukin-6, PTX3 is formed at the site of inflammation. It has been suggested that PTX3 plays the same role in the periphery as CRP does in the circulation. PTX3 may thus represent a rapid marker of local inflammation [11]. PTX3 is synthesized by monocytes and macrophages, vascular endothelial cells, fibroblasts and smooth muscle cells in response to inflammatory stimulants such as tumor necrosis factor-α, interleukin-1 and lipopolysaccharides [12,13]. As PTX3 is synthesized directly by cells that are involved in atherosclerosis, an increased level of this protein reflects an increased vascular inflammatory status. Therefore, it could be a more specific marker for the development of atherosclerosis and for prediction of progress in cardiovascular diseases [14,15]. 

Neprilysin, which is also referred to as neutral endopeptidase (NEP), is a membrane-bound, zinc-dependent endopeptidase also known as membrane metallo-endopeptidase (MME). In the circulation, neprilysin is a soluble circulating enzyme, and it is also present on the plasma membrane of neutrophils [16]. Neprilysin is responsible for natriuretic peptide degradation. Both atrial natriuretic peptide (ANP) and B-type natriuretic peptide (BNP) are hormones that are released when the atrium or chamber of the heart is stretched out [17]. The levels of neprilysin increase during poor renal function and in individuals suffering from heart disease. Inhibition of neprilysin increases levels of ANP, BNP, C-type natriuretic peptide (CNP) and bradykinin, which leads to vasodilation and decreases systolic blood and pulse pressure; furthermore, it has been shown to reduce mortality in heart failure patients (the Paradigm study [18]). Neprilysin inhibitors are now registered as a treatment for heart failure [19,20,21], and because of neprilysin’s ability to break down bradykinin, it may also be an interesting biomarker in STEMI. In this study, we aimed to investigate the dynamic changes in these biomarkers in STEMI patients. We analyzed samples collected at three different time points, and we followed the study group for 6.5–8.2 years to be able to study long-term survival, which is not common in previously published studies. 

## 2. Materials and Methods

### 2.1. Study Participants 

Blood samples from 165 patients suffering from acute ST segment elevation myocardial infarction (STEMI) were collected from the antecubital vein, into 3 mL sodium citrate vacutainer tubes. Samples were collected before (hereafter referred to as the acute samples), 1–3 days after, and 3 months after percutaneous coronary intervention (PCI) between December 2010 and August 2012 at the Department of Cardiology, Örebro University Hospital. As this study was performed as a sub-study of the multicenter TASTE study (Thrombus Aspiration in ST Elevation myocardial infarction in Scandinavia), the patients were also randomized to either have a manual thrombus aspiration performed or not in conjunction with their PCI procedure. The study design and patient selection criteria for the TASTE study have been previously published [22,23]. Since there were not complete data on all patients for all different biomarkers and all demographic data, the exact number of individuals included in each analysis is stated for each figure and table.

The control group consisted of 40 healthy donors at the Blood Center at Örebro University Hospital, aged between 18 and 65 years (mean 44 years, 42% women), from whom citrated blood was collected in conjunction with their blood/plasma donation. 

All subjects gave their informed consent to participation. The study was approved by the Regional Ethical Review Board in Uppsala, Dnr 2010/277 and Dnr 2010/294.

The original TASTE study was a multicenter study. A problem with multicenter studies is that there may be inter-hospital variations in blood sampling that are hard to identify. To minimize this risk, we only included patients and blood donors from Örebro. We also did a power calculation based on a difference between the groups of 20%, a two-sided *p*-value of 0.05 and a power of 0.80. As we expected a significant variation within the groups, we used a standard deviation of 30%. The calculation based on these figures resulted in a minimum group size of 36 individuals per group. The study included 165 patients and 40 controls, which is higher than the power estimate.

### 2.2. Blood Sampling and Laboratory Analysis

The collected venous blood samples were centrifuged at 2000× *g* for 10 min prior to laboratory testing. The plasma was divided into aliquots and stored at –80 °C for analyses, which were not performed immediately.

High-sensitivity CRP (reagent from Abbott Laboratories, Abbott Park, IL, USA) was analyzed using a BS380 instrument (Mindray, Shenzhen, China). The total analytic coefficient of variation (CV) for CRP was 1.5% at 2.5 mg/L. High-sensitivity troponin I was measured on an Architect ci8200 (Abbott Laboratories, Abbott Park, IL, USA) with reagents from the same manufacturer. The assay had a limit of detection of 2 ng/L and a 10% CV at 5 ng/L.

PTX3 and neprilysin were analyzed by sandwich ELISAs from R&D Systems (Minneapolis, MN, USA, products DY1826 and DY1182, respectively) according to the manufacturer’s instructions, and the absorbance was measured in a Spectra Max 250 (Molecular Devices, Sunnyvale, CA, USA). The ELISAs had total CVs of approximately 6%. All assays were performed blinded to clinical information. For statistical analysis, neprilysin values below the limit of quantification (100 pg/mL) were entered as 100 pg/mL. 

### 2.3. Statistics

Statistical analysis was performed using GraphPad Prism 8 (GraphPad Software Inc., San Diego, CA, USA). For unpaired data, the non-parametric Kruskal–Wallis test was performed, and for paired data, the non-parametric Friedman test was used, followed by Dunn’s multiple comparison test for post-hoc comparisons. Statistical significance was assumed when *p* < 0.05 was obtained. Comparison of survival curves was performed using the log-rank test (Mantel–Cox method), and the non-parametric Spearman correlation was used for correlation analysis. 

## 3. Results

### 3.1. CRP, PTX3 and Neprilysin in STEMI Patients

We studied 165 patients admitted with STEMI recognized at first admission to the emergency department during the period December 2010-August 2012. According to the available documentation, 74 of these had a thrombus aspiration and 74 did not have a thrombus aspiration performed (data were missing for 17 patients). Of the 165 patients, 118 were still alive (76 males and 28 females) while 47 had died (29 males and 18 females) at the follow-up analysis in March 2019. Demographic and clinical features of the patients at admission can be found in Table 1. 

### 3.2. Differences in Biomarker Levels as Compared with Healthy Donors

**Figure 1:**
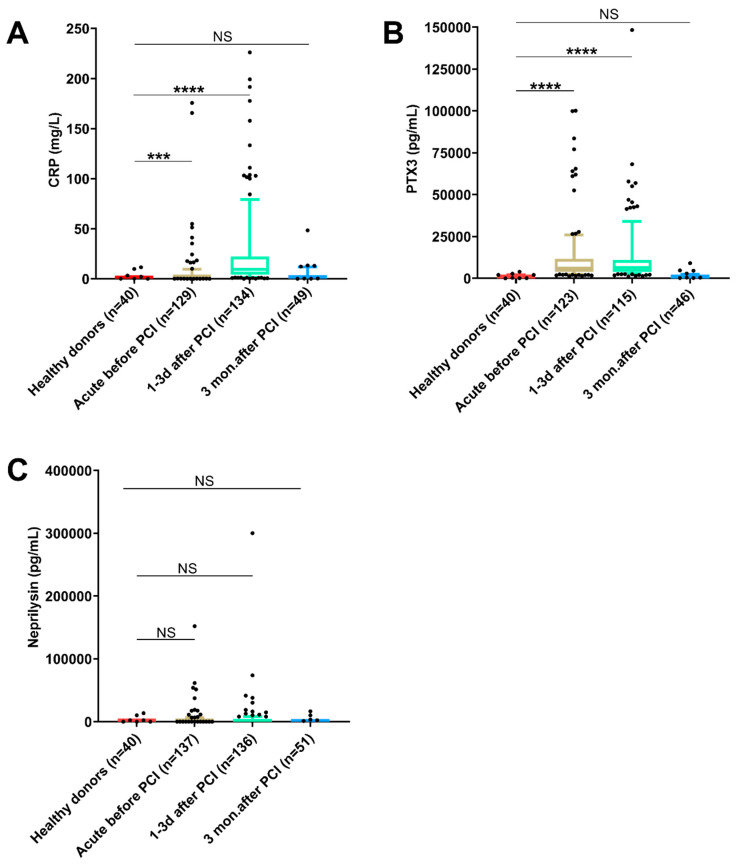
Plasma levels of (**A**) CRP, (**B**) PTX3 and (**C**) neprilysin in blood samples obtained from STEMI patients before, 1–3 days after and 3 months after PCI as compared with healthy donors. Statistical analyses were conducted using Kruskal–Wallis test followed by Dunn’s multiple comparison test; the whiskers show the 10th–90th percentile with all outliers. (NS = not significant, *** *p* < 0.001, **** *p* < 0.0001).

Figure 1 shows all available data and how the levels in STEMI patients at different time points compare with the levels in healthy donors. Quantitative values for the same dataset can be found in Supplementary Table S1. The plasma levels for CRP were significantly increased both in the acute samples and 1–3 days after PCI, with the highest levels 1–3 days after PCI, but at 3 months after PCI, the CRP levels were no longer higher than in healthy donors (Figure 1A). The same pattern was seen for PTX3, although high levels were observed here in both the acute and 1–3 days samples (Figure 1B). For neprilysin, there were no significant differences between the healthy donors and any of the three time points analyzed; however, notable inter-individual differences were observed, both in patients and healthy donors (Figure 1C and Supplementary Table S1).

### 3.3. Differences in Biomarker Levels between Patients with Patent and Occluded Culprit Vessels

According to the study protocol, the patients were categorized into two groups, one including patients with a patent (partially open) culprit vessel, and the other group containing those who had a totally occluded culprit vessel before PCI [24]. We therefore investigated whether the levels of these biomarkers differed between these two groups of patients, for the cases where reliable notifications regarding this were available. However, no significant differences were found for any of the time points investigated (see Supplementary Figure S1).

### 3.4. Differences in Biomarker Levels between Patients with and without Thrombus Aspiration 

As the objective for the TASTE study was to investigate the effects of thrombus aspiration in conjunction with PCI, we also investigated whether the levels of these biomarkers differed between patients who were randomized to having thrombus aspiration or not. However, there were no significant differences between patients with and without a thrombus aspiration (see Supplementary Figure S2).

### 3.5. Changes in Biomarker Levels at Different Time Points

**Figure 2:**
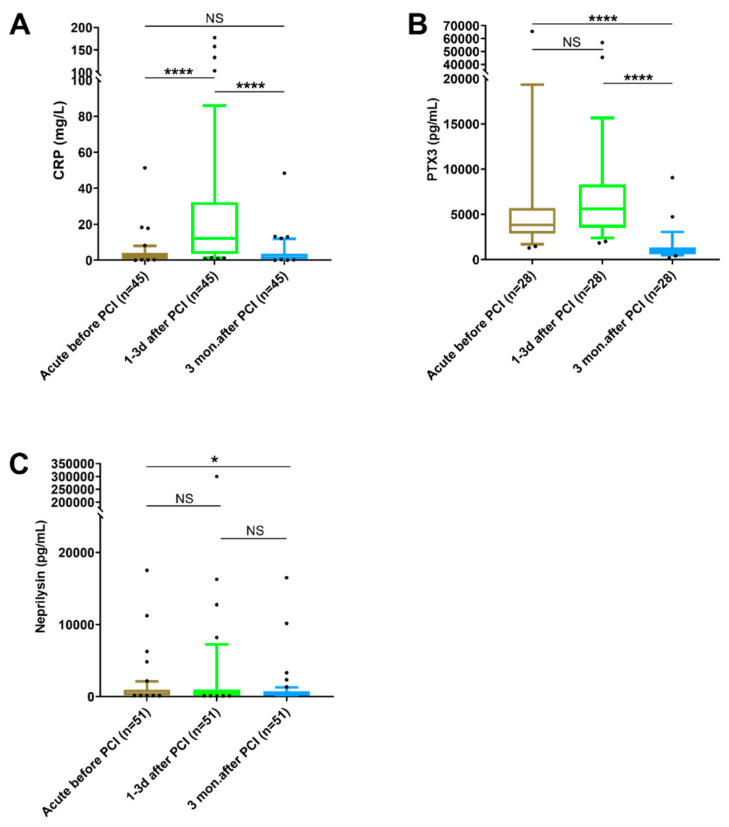
Plasma levels of (**A**) CRP, (**B**) PTX3 and (**C**) neprilysin in paired samples obtained at different time points during STEMI. Statistical analyses were conducted using the Friedman test followed by Dunn’s multiple comparison test, and data are shown as median and 10th–90th percentile with all outliers. (NS = not significant, * = *p* < 0.05, **** = *p* < 0.0001). Note that the y-axes contain breaks to help to also visualize the lower values.

As there were no significant differences between individuals with patent and occluded culprit vessels or with and without thrombus aspiration performed, we chose to analyze these together in the upcoming analysis of changes in levels for the biomarkers at the different time points. To prevent inter-individual variance affecting the results, only individuals for whom samples were available at all three time points were included, and paired analysis was used for the statistical testing. As Figure 2A shows, the plasma levels of CRP increased significantly from the acute samples to the samples taken 1–3 days after PCI, where the highest levels were found. At 3 months after PCI, the levels of CRP had decreased significantly again and were no longer different from those found in the acute samples before PCI (Figure 2A). For PTX3, the plasma levels were high in both acute and 1–3 days samples, with no significant difference between these. At 3 months after PCI, the levels were significantly decreased, both compared with the acute and the 1–3 days samples (Figure 2B). 

For neprilysin, a few individuals showed much higher levels than the others, but the only significant difference at the group level was between the acute and 3-month samples (Figure 2C). 

### 3.6. Correlation between Biomarkers 

**Figure 3:**
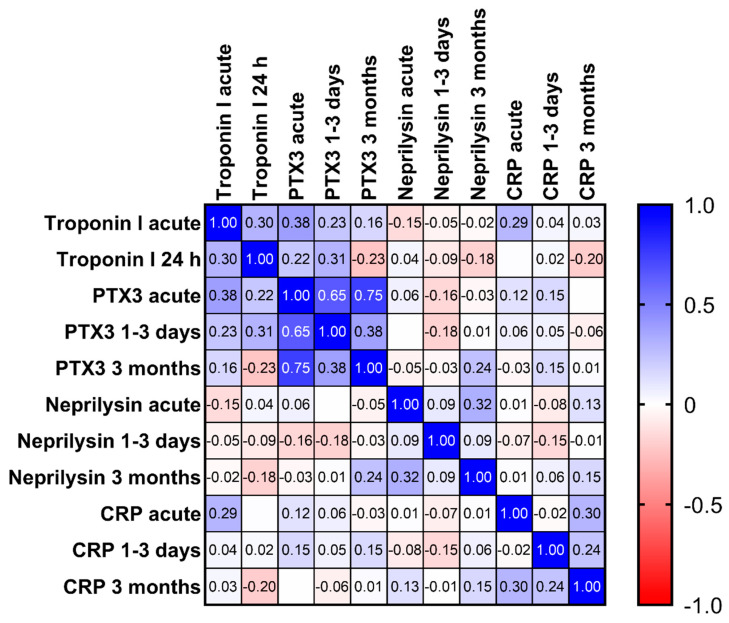
Heatmap showing Spearman r correlation coefficients between biomarkers and troponin at the different time points analyzed.

As shown in Figure 3, the Spearman correlation coefficients were generally weak, also for the same marker at different time points. The only exception was PTX3, where r was 0.65 and 0.75 when comparing acute values with those at 1–3 days and 3 months, respectively, and 0.38 between values at 1–3 days and 3 months. In these patients, troponin I was measured both in acute samples and after 24 h, but none of the markers showed strong correlations with troponin I at any of these time points; the highest r was 0.38 between PTX3 and troponin I in the acute samples. 

### 3.7. Survival Analysis of Patients with Elevated Levels of Biomarkers

**Figure 4:**
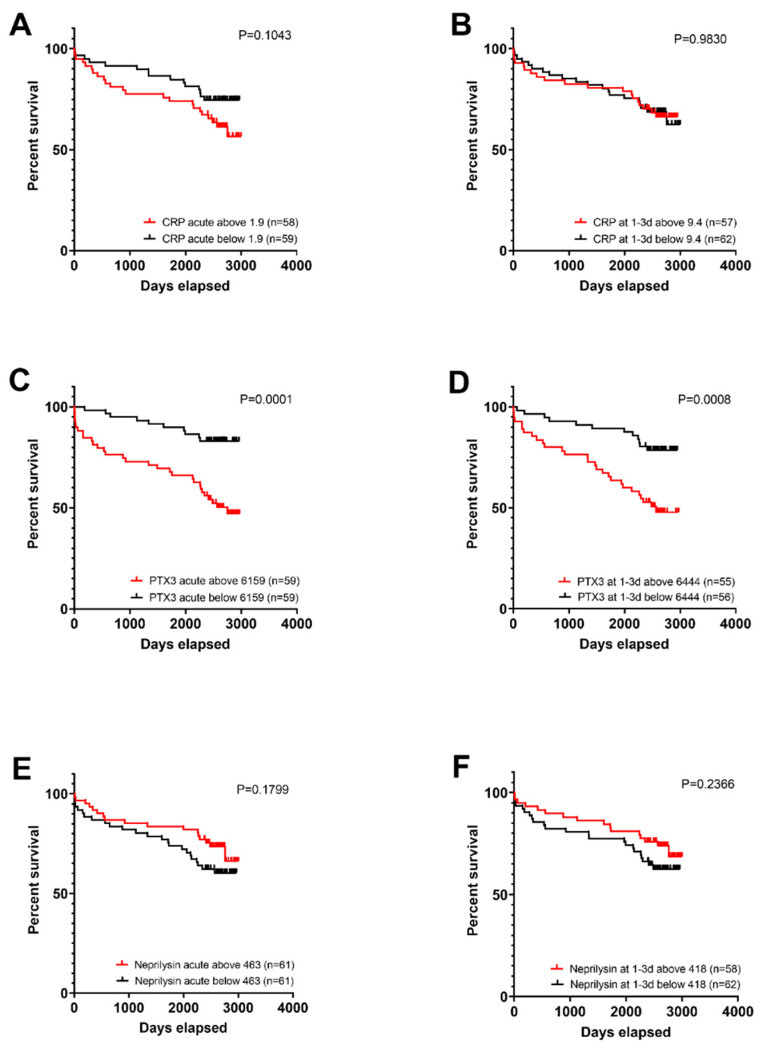
Kaplan–Meier curves comparing survival for patients with levels above and below the median value for (**A**,**B**) CRP, (**C**,**D**) PTX3 and (**E**,**F**) neprilysin. Curves in (**A**,**C**,**E**) are for acute samples, while (**B**,**D**,**F**) are for samples collected 1–3 days after PCI. Survival analysis was performed using the log-rank test (Mantel–Cox method).

To investigate whether biomarker levels showed any association with patient survival, we calculated the number of days between inclusion in the study and death (or days between inclusion and extraction of data for patients still alive in March 2019). The mean follow-up for all patients was 2202 days, with a range of 2381–3000 days of event-free survival. We then divided the patients into groups with biomarker levels above or below the median level of the marker at that time point. From these data, we created Kaplan–Meier curves, which can be seen in Figure 4. We performed testing both for acute samples and samples taken 1–3 days after PCI, as blood sampling at these two time points is most likely possible for the majority of patients in a clinical setting. The survival of the two groups was compared using the log-rank (Mantel–Cox) test. We found no significant differences between compared groups for neprilysin or CRP, but for PTX3 significant differences were observed both for acute and 1–3 days after PCI samples (Figure 4C,D). We also tested the construction of a survival plot with a cutoff at the upper reference limit (mean + 2SD) calculated from our 40 healthy blood donors, but as very few patients were below this limit for PTX3 (n = 11 in acute and n = 9 after 1–3 days) and very few patients were above this limit for neprilysin (n = 12 in acute and n = 13 after 1–3 days), this did not seem to be a feasible approach for these markers. For CRP, the groups were more evenly sized, and here a significant difference was observed in the acute samples (*p* = 0.0476), but not in samples collected 1–3 days after PCI (*p* = 0.6989).

## 4. Discussion

In this study, we analyzed dynamic changes in one well-established (CRP) and two more novel (PTX3, neprilysin) biomarkers in acute myocardial infarction.

We found that plasma levels of CRP and PTX3, but not neprilysin, were significantly elevated as compared with healthy donors. However, there were obvious differences in kinetics for the different markers. Unfortunately, only a small proportion of the 165 patients were investigated at all three time points, as the others were from other regions and thus had their follow-up visit at 3 months elsewhere. This reduces the strength of the analysis at three months, but we still believe it is more correct to do so, considering the high variability between individuals.

The acute-phase reactants CRP and PTX3 are both members of the pentraxin family. It has been known for decades that CRP increases during myocardial infarction and that the magnitude is related to infarction size [25]. Today, CRP is a well-established biomarker associated with inflammation in cardiovascular disease. In accordance with previous findings [26,27,28], we observed that PTX3 had faster kinetics than CRP, with high values found already in the acute samples. In contrast to CRP, PTX3 is produced locally at the site of tissue trauma and inflammation. Endothelial cells, dendritic cells and monocytes rapidly produce and release PTX3. Furthermore, neutrophils can instantly release granule-stored PTX3 [29]. This probably explains the rapid kinetics of circulating PTX3. 

PTX3 showed peak levels already at arrival, which suggests that PTX3 is related to the underlying disease and is not a consequence of the PCI intervention. However, as PTX3 was normalized 3 months after the PCI, it seems that the intervention helped to reverse the process leading to increased levels in the acute samples. There are previous studies that report increased levels of PTX3 in acute samples [27,28]. Others report increased PTX3 after PCI [9,30,31], and some report a reduction 1–3 days after PCI [9,27], but we have analyzed all three time points, which enables direct comparisons of the differences. 

In our study, we found a weak correlation between PTX3 and troponin I (r = 0.31), but there is no significant correlation between CRP or neprilysin and troponin I. Other studies have reported similar correlation coefficients between PTX3 and troponin T or I [9,31]. PTX3 more directly reflects the local inflammatory response in the injured vessel wall during STEMI [14]. This may explain the higher correlation between PTX3 and troponin than between CRP and troponin, although the correlation for PTX3 was still rather weak. 

Neprilysin is a biomarker involved in the regulation and degradation of vasodilator peptides. Our study found that levels of neprilysin in STEMI patients were not significantly elevated compared with healthy donors, although a few individuals, both patients and healthy controls, showed higher values. As the power calculations for the sub-study were not based on neprilysin data, a reason for this may be that the study was underpowered in that regard. However, similar findings were obtained in another study in STEMI patients, which reported that neprilysin serum levels did not change significantly during the first four hours following reperfusion, but that some individuals showed high values. These individuals were extensively studied regarding demographics and a number of different biological markers such as creatinine, hemoglobin, CRP, leucocytes and interleukin-6, and with cardiac magnetic resonance, but the researchers concluded that neprilysin levels did not reflect myocardial damage or inflammation [32]. When analyzing our patients with data from all time points, however, we observed a significant decrease in neprilysin after 3 months. This will reduce the breakdown of natriuretic peptides. This may in theory have a favorable effect on heart function similar to the use of neprilysin-inhibiting drugs. The mechanisms and relations between this finding and kidney and heart function after a STEMI might be an interesting subject for future studies. 

Interestingly, higher than median levels of PTX3 showed a clear association with future long-term mortality, both for acute samples and 1–3 days after PCI samples. There are several other studies suggesting that PTX3 might have value as a prognostic marker after PCI [9,30,31,33]. An early rise in PTX3 might also be beneficial, as rapid evaluation and treatment of MI is important for outcome. However, as PTX3 (in common with CRP) is a relatively non-specific marker, increased levels are seen in a larger number of inflammation diseases and infections [15]. The timing of sampling and selection of patients are therefore potentially very important for future evaluation of this potential. 

A limitation of the study was that the ethical permit only allowed for registering the age and sex of the healthy blood donors. Thus, we could not make adjustments for other factors such as smoking status or physical activity.

## 5. Conclusions

Neprilysin is not generally elevated during STEMI, although a few patients showed very high levels. Furthermore, our data confirm the differences in kinetics between the two pentraxins CRP and PTX3, with PTX3 levels being high already in the acute samples while the peak for CRP came 1–3 days after PCI. The present study further strengthens the suggestion in previous publications that PTX3 might be of interest for future studies as a potential predictor of long-term mortality in STEMI patients. Prospective studies based on the current knowledge in this area would be of great interest.

## Figures and Tables

**Table 1:** Baseline demographic, clinical and procedural characteristics of the 165 STEMI patients.

Age, Years (mean, range)	69 (43–94)
Female/male	46/104
Prior myocardial infarction	17%
Smoking, current	34%
BMI (kg/m^2^).(mean, range)	27.1 (16.3–39.1)
Hyperlipidemia (according to patient anamnesis)	52%
Diabetes mellitus (any type)	18%
(according to patient anamnesis)	46%
Unstable angina	10%
PPT (10^3^/µL, mean ±SD)	228 ± 62
WBC (10^3^/µL, mean ±SD)	10.8 ± 4.1
Lesion type	
Occluded	70%
Patent (partially open)	30%
Medication (at admission before PCI)	
ASA (only)	22%
ASA+clopidogrel	2%
Betablockers	27%
Statins	26%

BMI—body mass index; PPT—platelet particle concentration; WBC—white blood cell count; ASA—acetylsalicylic acid.

## Data Availability

The datasets used and/or analyzed during the current study are available from the corresponding author on request. This will in most cases also require an ethical permit.

## Supplementary Materials

The following are available online at https://www.mdpi.com/article/10.3390/biomedicines10020275/s1.
