# Supplementary material for: Dynamic Changes in Pentraxin-3 and Neprilysin in ST Segment Elevation Myocardial Infarction
Source: Biomedicines. 2022 Jan 26;10(2):275. doi: 10.3390/biomedicines10020275 (PMC8869300; doi:10.3390/biomedicines10020275)

Supplementary material to:

Dynamic changes in pentraxin-3 and neprilysin in ST segment elevation myocardial infarction

Rahel Befekadu[1,2], Magnus Grenegård[2], Anders Larsson[3], Kjeld Christensen[4], Sofia Ramström[2,5]

[1]Department of Laboratory Medicine, Section for Clinical Immunology and Transfusion medicine, Örebro University Hospital, Örebro, Sweden.[2]Cardiovascular Research Centre, School of Medical Sciences, Örebro University, Örebro, Sweden.
[3]Department of Medical Sciences, Uppsala University, Uppsala, Sweden.
[4]Karlstad Central Hospital, Karlstad, Sweden.
[5]Department of Clinical Chemistry, and Department of Biomedical and Clinical Sciences, Linköping University, Linköping, Sweden

Supplementary Table S1: quantitative data on levels of CRP, pentraxin-3 and neprilysin in the study population and the healthy donors.

Marker	Time point	Median	$25^{th}/75^{th}$ percentile	95% confidence interval
**CRP (mg/L)**	Acute (n=129)	1.9	0.7 / 4.3	1.5-2.6
	1-3 days after PCI (n=134)	9.4	4.2 / 22.1	8.2-12.7
	3 months after PCI (n=49)	1.4	0.6 / 3.6	0.8-2.1
	Healthy donors (n=40)	0.6	0.3 / 1.0	0.4-0.8
**PTX3 (pg/mL)**	Acute (n=123)	6159	3797 / 11594	4953-7407
	1-3 days after PCI (n=115)	6444	3858 / 10865	5613-7255
	3 months after PCI (n=46)	940	624 / 1367	708-1196
	Healthy donors (n=40)	821	563 / 1274	697-1146
**Neprilysin (pg/mL)**	Acute (n=137)	463	244 / 1031	351-670
	1-3 days after PCI (n=136)	418	141 / 1000	304-543
	3 months after PCI (n=51)	302	143 / 748	243-449
	Healthy donors (n=40)	513	237 / 915	352-811

Supplementary Figure S1: Plasma levels of (A) CRP (B) PTX3 and (C) neprilysin in blood samples obtained from STEMI patients before, 1-3 days and 3 months after percutaneous coronary intervention (PCI), comparing patients with patent (partially open) culprit vessel to those having an occluded culprit vessel. Statistical analyses were conducted using Kruskal Wallis test followed by Dunn´s multiple comparison test, the whiskers show the 10th–90th percentile with all outliers. (NS= not significant).

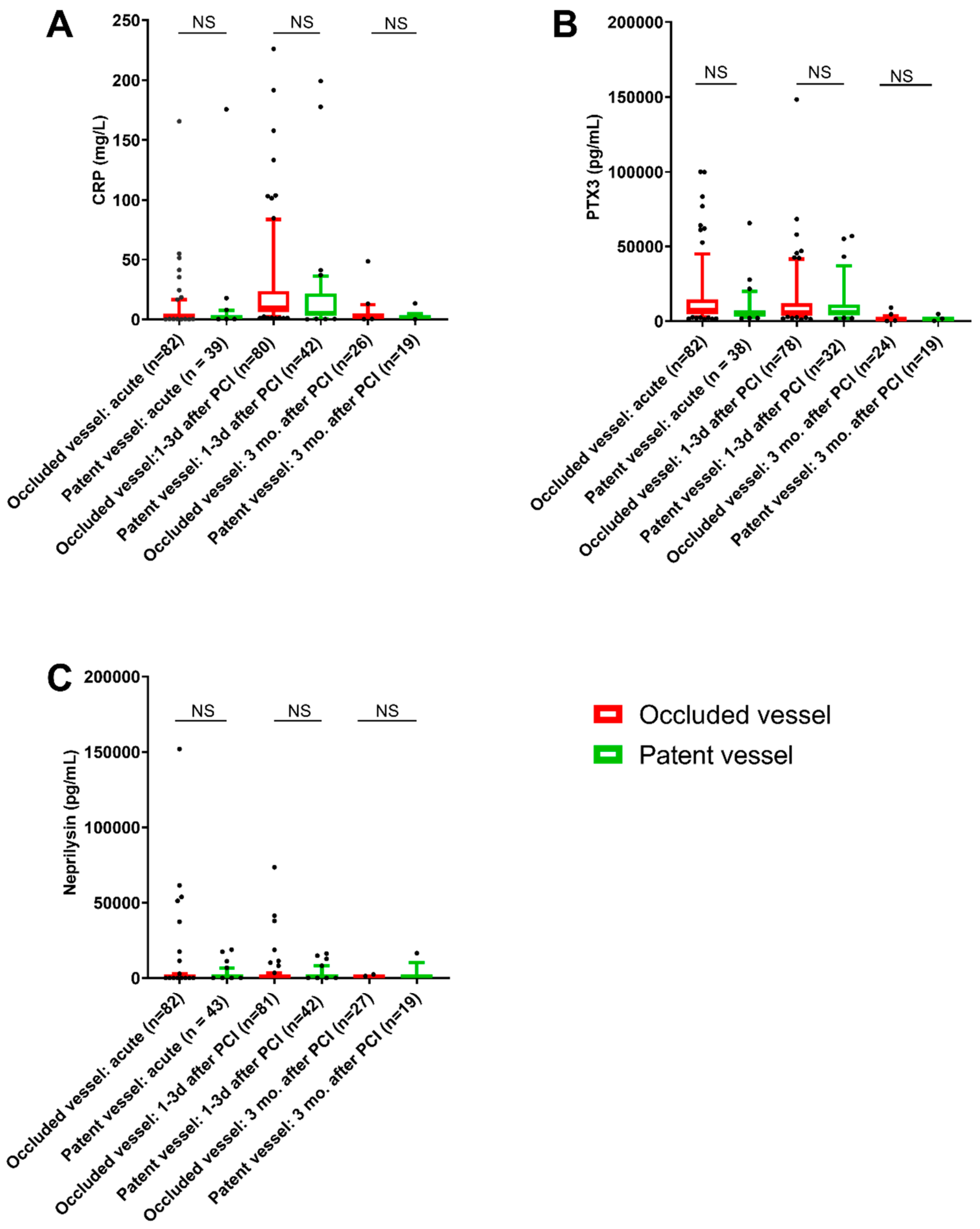

Supplementary Figure S2: Plasma levels of (A) CRP (B) PTX3 and (C) neprilysin in blood samples obtained from STEMI patients before, 1-3 days and 3 months after percutaneous coronary intervention (PCI), comparing patients where thrombus aspiration was performed to those who did not receive any thrombus aspiration. Statistical analyses were conducted using Kruskal Wallis test followed by Dunn´s multiple comparison test, the whiskers show the 10th–90th percentile with all outliers. (NS= not significant)

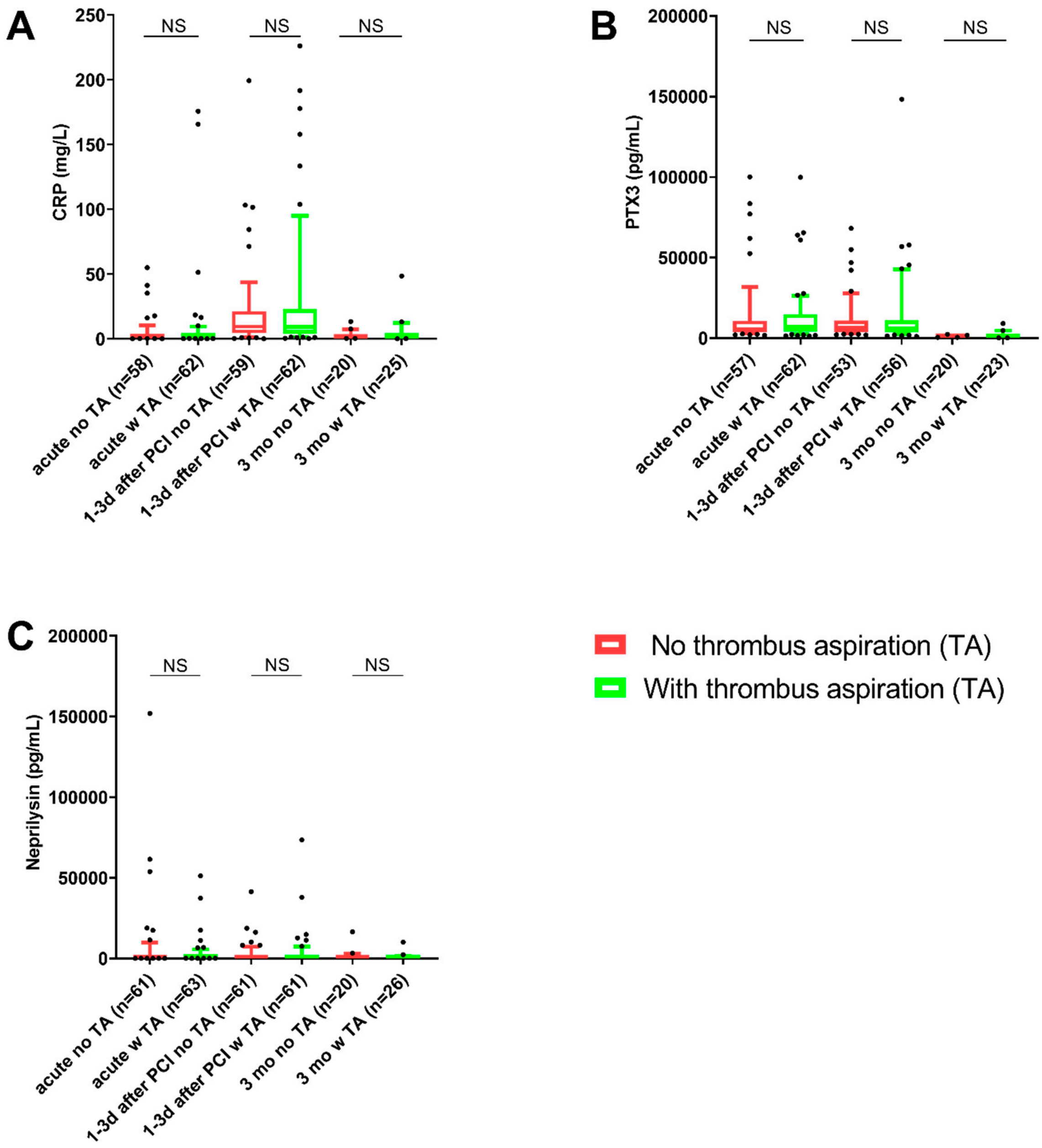

Supplement: Supplementary file 1 [file biomedicines-10-00275-s001.zip › biomedicines-1518643-supplementary.pdf]
